# Passive high explosive neutron inspection (PHENIX): a new method to confirm the presence or absence of high explosives for nuclear treaty verification

**DOI:** 10.1038/s41598-024-58839-5

**Published:** 2024-04-29

**Authors:** David L. Chichester, James T. Johnson, Jay D. Hix, Edward H. Seabury

**Affiliations:** Idaho National Laboratory, 2351 N. Boulevard, Idaho Falls, ID 83415 USA

**Keywords:** Engineering, Applied physics

## Abstract

Advanced instruments and methods need to be developed now to create a technical basis to support the negotiation of future nuclear arms control treaties. One new capability that is anticipated is the ability to confirm either the declared presence or declared absence of high explosive (HE) material in the presence of special nuclear material (SNM). Towards this goal, Passive HE Neutron Inspection (PHENIX) has been developed and demonstrated as a method for confirming the presence or absence of HE in the presence of plutonium. The method exploits the inherent presence of neutrons associated with the decay of plutonium as an internal probe source for performing prompt gamma-ray neutron activation analysis (PGNAA), searching for the presence of HE as revealed by the emission of characteristic gamma rays following neutron absorption in hydrogen and nitrogen which are building blocks of present-day, military-grade HE. Tests using stoichiometrically-correct hemishells of mock HE with plutonium show that a system can be expected to positively confirm the presence or absence of these signatures, supporting determination of HE presence or absence with Pu, in a few hours. To protect other potentially sensitive gamma-ray signatures from a treaty accountable item, an analog information barrier has been conceptualized and tested which physically prevents the collection of gamma-ray spectral data outside of user selected energy windows strategically chosen to view only narrow spectral regions corresponding to the hydrogen (2223.2 keV) and nitrogen (9807.2 keV, 10,318.2 keV, and 10,829.2 keV) PGNAA signatures.

## Introduction

In contrast with the New Strategic Arms Reduction Treaty^1^, future arms control agreements may require more intrusive technical verification measurements to support verifying objects presented for inspection either are or are not nuclear warheads. In this context, passive methods for verifying the presence or absence of high explosives (HE) in the presence of plutonium in sealed containers and/or warheads can be used as one part of a verification regime for confirming a treaty accountable item (TAI) either is (HE present) or is not (HE absent) a nuclear warhead^2–5^. Such techniques should be suitable for on-site inspections inside nuclear warhead storage facilities, as well as in portal monitors located at facility entrance/exit points. This capability, a confirmatory verification technology, is needed to provide options to negotiators of future arms control treaties. It can support assessments that confirm an item is a warhead, verify the declared operational readiness of a warhead, verify that a TAI belongs to a certain class of TAIs, verify that special nuclear material (SNM) has been separated from HE, and verify that warhead dismantlement has occurred. An important aspect of this need is that technical solutions must protect sensitive design information.

Today there are no accepted measurement technologies that use passive methods for determining the presence or absence of HE material with SNM in sealed containers and/or warheads. (Passive in this sense means that no external radiation sources, e.g., neutrons or x rays, are used to induce signatures in TAIs.) One approach that has been proposed for this problem is to use the difference in count rates between different rows of a polyethylene-moderated helium-3 neutron detector, this is called the “row ratio” method^6^. The row ratio method was originally developed to assess the presence and impacts of moderating materials around plutonium in safeguards coincidence counting but its use can be extrapolated for assessing the presence of HE near Pu. Related to this is a detector called “SSPN10”, a multilayer neutron detector constructed by the Research Institute of Pulse Technique, Moscow, Russia^7^. This detector provides neutron energy spectral information for incident neutrons in a five-channel histogram and is intended for use in a template-matching scheme. In general, these detectors measure the ratio of slow neutrons to fast neutrons emanating from an item or container and can be used to infer the absence of HE with Pu in some cases.

As a complement to these approaches, a new passive HE neutron inspection (PHENIX) method based on prompt gamma-ray neutron activation analysis (PGNAA) has been developed and tested to meet this need. The PHENIX approach uses prompt gamma-ray neutron activation analysis (PGNAA) to measure the characteristic neutron-capture gamma rays and associated signatures from nitrogen, hydrogen, and potentially other key components found in military HE (see Table 1)^8–10^. (For HE presence verification, this signature serves as a presumptive screening technique, since an elementally-correct mock (fake) HE would provide the same signature as real HE. For HE absence verification, this signature serves as a confirmatory technique). Passive in this sense means that no external radiation source is used to induce the PGNAA signatures, in contrast with measurements where an external neutron source such as ^252^Cf or an electronic neutron generator are used. The use of PGNAA with an external neutron source for HE detection, identification, and characterization is well known^11,12^. This approach relies on the use of the neutrons naturally produced in the decay of ^240^Pu or other actinide isotopes, and any additional neutrons inherently produced through assembly multiplication.

**Table 1:** Summary of density and elemental composition for several HE materials^8–10^.

Material	Formula	Nominal density, g cm^−3^	Elemental composition, wt. %				
			C	H	N	O	Other
Comp-B	C_2.03_H_2.64_N_2.18_O_2.67_	1.71	24.3	2.7	30.4	42.6	–
HMX	C_4_H_8_N_8_O_8_	1.89	16.2	2.7	37.8	43.2	–
PBX-9501	C_1.47_H_2.86_N_2.60_O_2.69_	1.84	17.7	2.9	36.4	43.0	–
PBX-9502	C_2.30_H_2.23_N_2.21_O_2.21_Cl_0.038_F_0.13_	1.90	27.6	2.2	31.0	35.4	Cl: 1.3 F: 2.5
PBX-9503	C_2.16_H_2.28_N_2.26_O_2.26_Cl_0.038_	1.88	26.6	2.4	32.5	37.1	Cl: 1.4
PETN	C_5_H_8_N_4_O_12_	1.76	19.0	2.6	17.7	60.7	–
RDX	C_3_H_6_N_6_O_6_	1.8	16.2	2.7	37.8	43.2	–
TATB	C_6_H_6_N_6_O_6_	1.88	27.9	2.3	32.6	37.2	–

Prior work exploring aspects of the PHENIX approach has been presented elsewhere. A joint research effort between the Massachusetts Institute of Technology and Lawrence Livermore National Laboratory concluded that “…passive detection of the expected high-energy gamma signal is not feasible…,” attributing the difficulty to “… competing backgrounds produced by neutron-source interactions with surrounding materials”^13^. In contrast, a computational study by researchers from the Institute of Applied Physics and Computational Mathematics, China, bolsters the theoretical feasibility of the PHENIX approach^14^. That paper concluded that the passive PGNAA measurement approach should work. While that effort was purely computational and did not contain experimental verification measurements, and although their simulation work failed to properly address the impact of background interferences in the PGNAA measurement, the conclusion was still compelling.

## Methods

To demonstrate the feasibility of using PHENIX measurements for HE absence or presence verification a set of laboratory experiments was performed to study the PHENIX signatures, look for interferences, and assess signal intensities to establish bounding limits on detection sensitivity and measurement times. The tests used a metallic disk of plutonium (to mimic the application and to ensure the presence of relevant gamma rays) coupled with extra ^252^Cf sources, to boost the neutron signature. Gamma-ray emissions from this assembly were then measured using sets of mock HE hemishells of varying thickness and composition.

### The PGNAA signatures

As seen in Table 1, typical high-energy–density explosives relevant for arms control verification contain the elements C, H, N, and O. The PHENIX approach focuses on measuring the gamma-rays emitted from the capture of thermal neutrons in hydrogen and nitrogen. For hydrogen, the reaction ^1^H(n,γ)^2^H produces a characteristic gamma-ray at 2223.2 keV. For nitrogen, the reaction ^14^N(n,γ)^15^N produces many gamma rays, including strong emissions at 1884.8 keV (18.8%), 3677.4 keV (14.5%), 4508.7 keV (16.7%), 5269.2 keV (29.9%), 5297.8 keV (21.2%), 5533.4 keV (19.6%), 5562.1 keV (10.7%), 6322.4 keV (18.2%), and 10,829.1 keV (14.3%). In practice the highest energy emission at 10,829.1 keV often produces the signal with the best signal-to-noise ratio. In addition to this gamma ray, spectroscopy also allows interpretation of the single escape (SE) artifact at 10,318.1 keV and the double escape (DE) artifact at 9807.1 keV. As shown below, while the most intense nitrogen PGNAA gamma-rays occur at lower energies, their spectroscopic interpretation suffers due to the presence of competing gamma-rays and their associated artifacts (Compton continua and escape peaks), most notably due to the presence of iron (e.g., at 7631.1 keV, 7645.5 keV, and several lower energies) which will likely be present in the warhead itself, handling gear, and nearby structures.

### Test items

**Figure 1:**
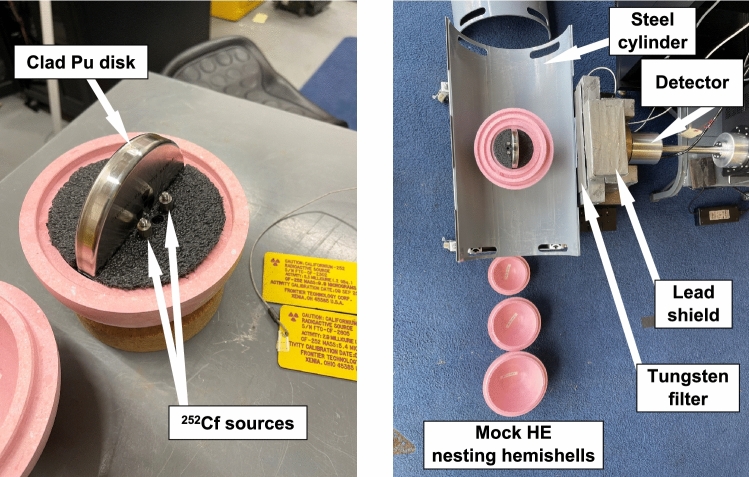
Photographs of the test set up. Close-in photograph (left) showing the Pu disk and Cf sources used for the test item and wider photograph (right) showing the test item within half of three layers of the nested 900-19 hemishells, placed in the bottom half of the steel cylinder, with the detector and its shielding on the right.

For SNM a doubly-encapsulated disk of metallic plutonium was used for the basis of the test item. The outer diameter of the disk was 73.6 mm and its thickness was approximately 2.9 mm. Considering self-attenuation, this thickness is essentially “infinitely thick” for the 129.3 keV gamma rays from ^239^Pu and the 59.5 keV gamma rays from ^241^Am^15^. The total mass of plutonium was 195.2 g, its isotopic composition was: 0.0131 wt.% ^238^Pu, 93.9 wt.% ^239^Pu, 5.91 wt.% ^240^Pu, 0.104 wt.% ^241^Pu, and 0.0409 wt.% ^242^Pu. The disk also contained 0.1 g of ^241^Am. Using methods proposed by Fetter et al.^16^, the neutron emission rate from this disk was estimated to be 1.08 × 10^4^ neutrons s^−1^. To boost this in order to represent a more realistic level for this problem, two ^252^Cf sources were placed behind the disk, so that the total neutron emission rate was 3.08 × 10^5^ neutrons s^−1^, roughly equivalent to the emission rate expected from 5.5 kg of the same type of Pu. This corresponded to approximately 0.69 of a ‘significant quantity’ (SQ, 1 SQ = 8 kg) of plutonium, as defined by the International Atomic Energy Agency^17^. The assembly of Pu and Cf was held within a low-density foam half-sphere, orientating the plane of the Pu disk normal to the measurement system, with the Cf sources behind the Pu (Fig. 1).

In all measurements the test item was placed within a two-part steel cylinder, 6.4 mm thick, serving to mimic additional structure for a TAI or a storage container. In addition to measuring PGNAA gamma-rays from this bare PuCf assembly, measurements were taken with this assembly encased within two different sets of nesting shells of mock HE (Fig. 1). Each set included three pairs of 20-mm thick hemishells, with inner diameters of 120 mm, 160 mm, and 200 mm. One set (900-19) was made to mimic the elemental composition of high explosive PBX 9502, it had an atomic composition of C: 28.4%, H: 2.3%, N: 29.5%, O: 33.9%, F: 4.0%, and Cl: 2.0%, with a density of 1.67 ± 0.02 g cm^−3^. The other set (905-05) mimicked the elemental composition of the high explosive COMP-B, it had an atomic composition of C: 30.2%, H: 2.7%, N: 30.6%, and O: 36.6%, with a density of 1.64 ± 0.02 g cm^−3^. The masses of the 900-19 assemblies were 2031.8 g, 5351.8 g, and 10,336.0 g for total thicknesses of 20 mm, 40 mm, and 60 mm. The masses of the 905-05 assemblies were 1995.8 g, 5333.2 g, and 10,354.0 g for the same thicknesses.

### Measurement system and analog information barrier

Gamma-ray emissions for the experiments were measured using an n-type, high-purity germanium (HPGe) gamma-ray spectrometer (ORTEC, Oak Ridge, Tenn., USA) with an efficiency of 50% relative to a 3′′ × 3′′ NaI scintillator. The detector has a wide-range amplifier to allow it to operate up to 12,000 keV, permitting it to record the highest energy gamma ray from the PGNAA thermal capture reaction in nitrogen. The detector was surrounded with ~ 50 mm of lead on its sides, top, and bottom. Often, direct gamma-ray spectrometry of plutonium is challenging due to the high flux of lower-energy x-rays and gamma rays from the Pu and associated Am isotopes. To reduce this impact, 2.1 mm of tungsten was placed between the front face of the detector and the test area (Fig. 1).

**Figure 2:**
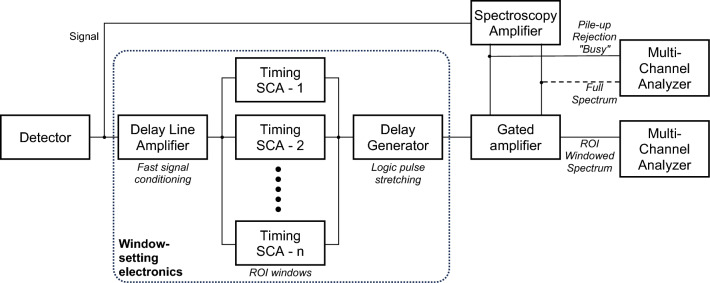
Schematic representation of the analog information barrier.

Direct gamma-ray spectrometry of a TAI can reveal sensitive information about the composition of SNM in the device, and possibly other information as well. To address this for arms control verification, spectrometry measurements are often planned to be used in conjunction with an “information barrier,” a combination of hardware and software technology that (a) only allows non-sensitive information to be revealed to the operators and (b) ensures that the revealed information accurately reflects the measured conditions^18^. To address the need for this in the PHENIX implementation described here, a new type of analog information barrier (aIB) was developed using discrete nuclear instrumentation module (NIM) electronics. A schematic representation of the aIB is shown in Fig. 2. The signal from the HPGe detector is split and sent (a) to a spectroscopy amplifier and (b) to a set of user-configurable single-channel analyzers (SCAs) that work to define region-of-interest (ROI) windows that limit the final observed spectrum. The amplified full spectrum signal is then split and sent (a) directly to a multi-channel analyzer (MCA) for calibration, certification, and authentication analyses, and (b) sent to a gated amplifier, which rejects signals not falling in the pre-defined ROI windows, after which the signal is also sent to an MCA for analysis.

The idea for using an analog, hardware-based information barrier (aIB) approach is inspired by a concept proposed by Buhler et al.^19^, where they incorporated an IB within the analog-to-digital conversion circuitry of an MCA at the chip level. The PHENIX approach differs by using simple, analog modules, with a goal of reducing susceptibility to the digital “side-channel attacks” identified by Buhler et al. in their approach. A key extra benefit of the approach outlined in Fig. 2 is that, while the pile-up rejection “busy” signal is sent to the MCA for the full spectrum analysis, it is not sent to the MCA for the ROI windowed spectrum. Thus, the true dead time for the measurement, and thus the true live acquisition time information, is lost and not available for analysis. Because of this, it is not possible to correlate gamma-ray signal intensities to true emission rates from the TAI, which therefore precludes the ability to infer isotopic information or masses about the Pu in the TAI. In our tests the deadtime (as determined from the full spectrum data) varied from 78.5% for the bare PuCf assembly, to 44.3% for the bare PuCf assembly with a 2.1 mm W filter, to 25.9% for the measurements of 3 shells of 905-05 plus the W filter. In practice, a host nation could also vary the thickness of the W filter without informing the inspecting party, adding additional protection against learning a measurement’s true live acquisition time.

During calibration, testing, and authentication and certification^20^, the operators would observe both the full spectrum and the ROI windowed spectrum using a non-sensitive calibration aid, such as a cylinder made of mock HE that contains a ^252^Cf source. With this they would be able to confirm the ROI windowed data matched the full spectrum, and verify the system can detect H and N. When a TAI is analyzed, the cable passing the full spectrum data from the spectroscopy amplifier to the MCA (the dotted line in the figure) would be physically removed, eliminating the ability of the operator to observe sensitive spectral information from the decay gamma-rays of Pu, Am, or other signatures which are not present in the ROI windows. This aIB concept presents several desirable attributes^21^ for authenticating an arms control measurement system, including: simplicity; ease of inspection; transparency (using all commercial components); the ability to randomly choose components from a pool of components; the ability to perform tests of the system against known, non-sensitive objects during a measurement sequence; having an open and closed model; using modular and minimally-functional parts; and having the ability to maintain continuity of knowledge of the system following initial authentication.

## Results

**Figure 3:**
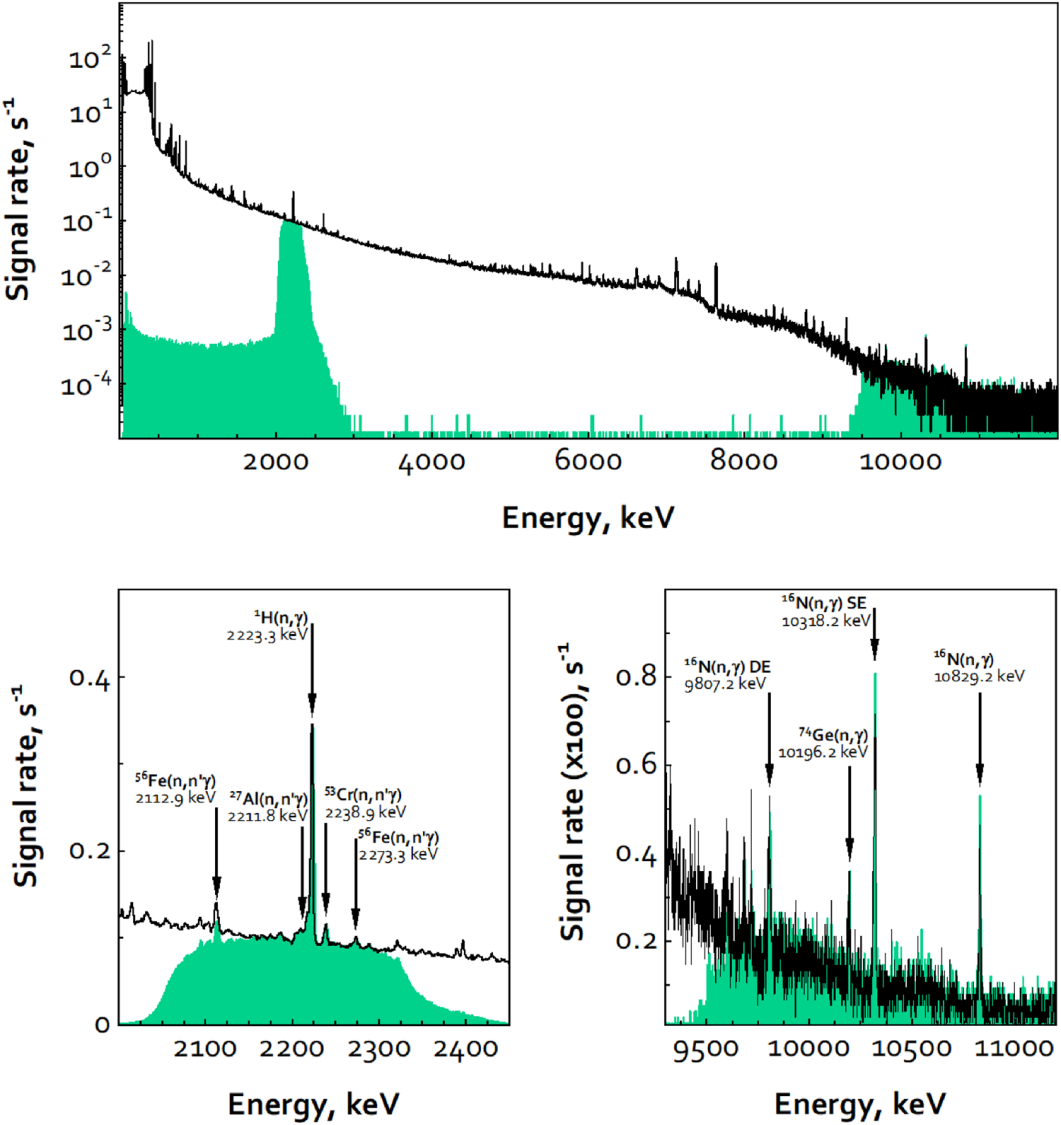
Gamma-ray spectrum from three shells (60 mm) of mock HE 905-05. The top plot shows the complete spectrum (black) and ROI windowed spectrum (green), the lower plots show the expanded and annotated regions for the hydrogen capture gamma ray (left) and the high-energy nitrogen capture gamma ray (right).

An example gamma-ray spectrum from the measurement of the full set of three mock HE 905-05 shells is shown in Fig. 3, including the full spectrum (top) and expanded views of the hydrogen and nitrogen regions of the ROI windowed spectrum (bottom). Spectra from mock HE 900-19 are similar to this. Many features are present in the full spectrum, including the gamma rays from isotopes of Pu and Am, and a variety of thermal-neutron capture gamma rays and inelastic neutron-scattering gamma rays from structural components in and near the test item. Long measurement acquisition times of greater than twelve hours were used to collect all spectra, to ensure good peak fitting and counting statistics. Recognizing that measurement times this long would not be practical, the data was reduced to counting rates, to allow scaling to different measurement durations.

**Figure 4:**
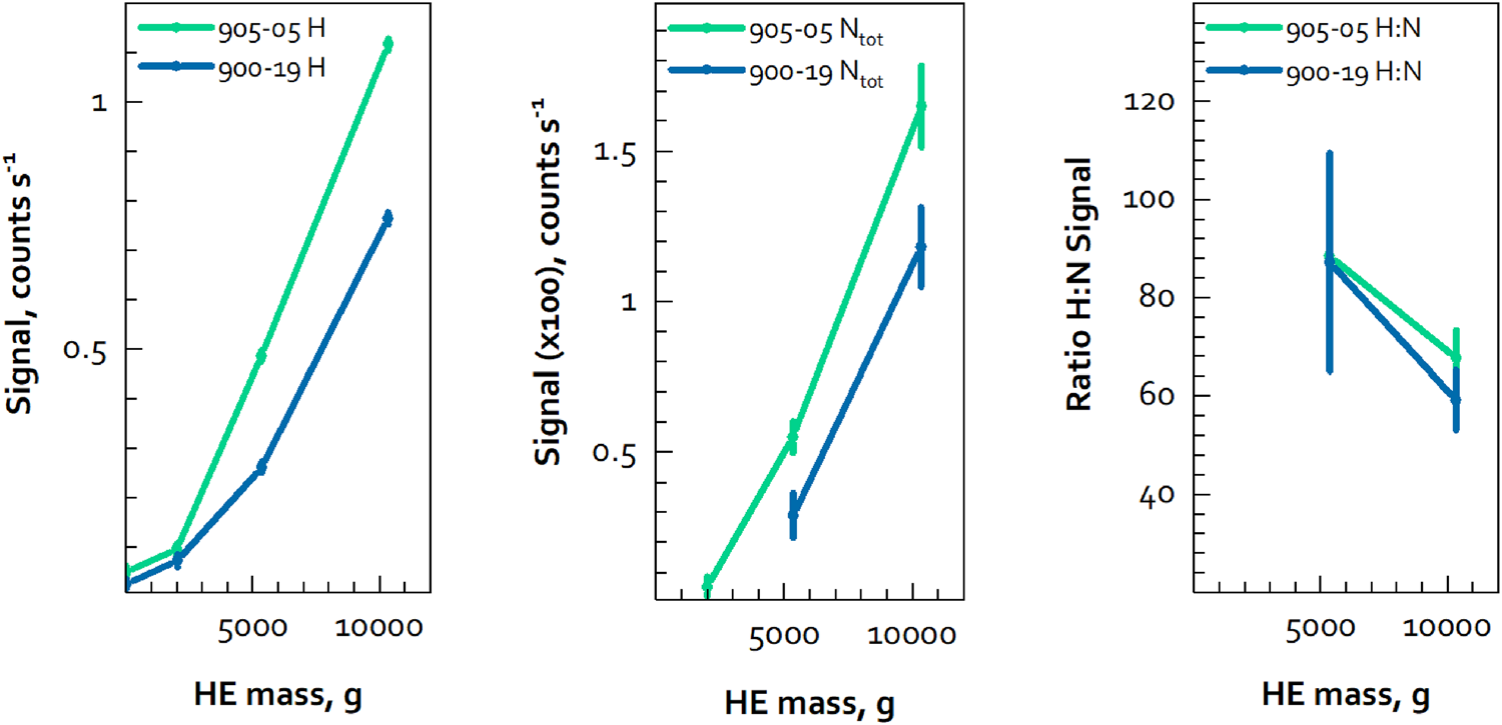
Signal intensity for H and N, and the H:N signal ratio.

Both sets of data show progressively weaker H and N signatures as the mock HE mass is reduced, as seen in Fig. 4. For the test cases used here the signal rates for the total nitrogen signature (the photopeak plus the SE and DE peaks) from one-shell of 900-19 are too weak for analysis.

## Discussion

It is clear that passive PGNAA measurements of hydrogen and nitrogen serve as strong signatures for the presence of HE (or elementally-similar mock material) in the presence of Pu, and that their absence serves as an equally strong indicator of the absence of nitrogen-bearing HE in the presence of Pu. While both are important, and hydrogen is the more intense signal to be measured, nitrogen provides the HE-specific fingerprint in this application. The question that remains is to consider the practical feasibility for using this in arms control for treaty verification.

**Figure 5:**
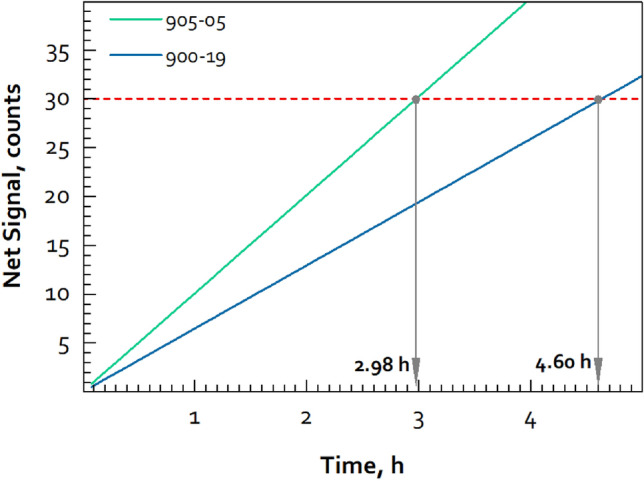
Net signal increase over time for the N SE peak, for one detector (this is for the two-shell configurations).

For peaks in the high-energy nitrogen window, empirical observations show that a net signal of approximately 30 counts, with a signal-to-noise (S:N) ratio of 0.1 or better, is needed to positively confirm the presence of a photopeak, or SE or DE peak. (In this measurement geometry, the SE peak is always the strongest of these three peaks and the first to become detectable above background.) These two criteria can be used to assess the measurement time needed to confirm (presumptively) the presence of HE (numerous other approaches could also be used). If we consider the cases with two shells, both mock HE materials met the S:N criteria. For net signal, the measurement geometry used here resulted in a net nitrogen SE signal rate of 0.0018 counts s^−1^ for 900-19 and 0.0028 counts s^−1^ for 905-05. These compete with a background signal of ~ 0.0005 counts s^−1^. A plot illustrating how these net signatures evolve over time is shown in Fig. 5. The time needed to reach a net signal of 30 counts for 900-19 is 4.60 h (5357 g), for 905-05 it takes 2.98 h (5533 g). If these two masses were accepted as the minimum detection limit needed for presumptive presence confirmation and true absence confirmation, then these times would be the measurement times needed for these two types of mock HE. Noting that the H signal is more intense than the N signal with the mock HE, the potential use of the H:N ratio could also be used as a secondary vote of confidence for confirming the presence of HE. Other spectral fiducials, such as the ^56^Fe(n,n′γ) or ^74^Ge(n,γ) lines, might also be chosen for this purpose.

For 900-19 the practical, absolute minimum detection limit is greater than the one-shell case used here, since that arrangement did not produce a measurable signature after 14.9 h. For 905-05, the one-shell configuration was measurable and the S:N (0.46) met the first criteria. However, the time needed for that arrangement to reach the 30-count criteria would be much longer, at 36.1 h. If a minimum detection limit of two-shells was again chosen as the criteria for confirming the absence of HE then the counting times shown above would again be valid. To reduce the measurement time it would be reasonable to consider using additional detectors. Using four detectors, still with a goal of a two-shell minimum detection limit, would allow the measurement to be performed in 1.15 h for 900-19 and 0.75 h for 905-05. These times are considered reasonable by experts in this field. Additional time might be needed for a measurement protocol to account for using a stand-off distance—in the work presented here the detector apparatus was at the surface of the test item. Also, additional margin might be desired to account for errors in positioning, or to add confidence to the results. Nevertheless, it is clear that passive PGNAA can be used in a reasonable time to presumptively confirm the presence or positively confirm the absence of HE in the presence of Pu for some types of future arms control treaty verification measurements.

Referring again to Fig. 3, the performance of the aIB is observed to completely mask spectral signatures from the full spectrum that are outside of the two ROI windows—meeting the goals for protecting sensitive information present outside of the ROIs. However, the energy regions outside of the ROI windows are not completely empty. The source for count events in these areas is not fully understood but is believed to be due in part to imperfect optimization of the gate windows in the “window-setting electronics” section shown in Fig. 2, leading to energy clipping of events from within the windows. Also worth noting is that the upper and lower energy edges of the ROI windows have a gradual roll off, rather than hard edges. This too is due to the analog nature of the counting system and associated time pick-off jitter. Optimization would likely also help improve this, to make these edges steeper, but it would not likely be able to completely eliminate the fall off. If a sensitive signal was observed to be present on one these shoulders, then the ROI window would need to be narrowed to ensure it was not present in the ROI windowed spectrum.

## Data Availability

The datasets generated during the current study are available from the corresponding author on reasonable request.
